# Effect of Remote Vital Signs Monitoring in People Receiving Long-Term Oxygen Therapy: Protocol for a Randomized Controlled Trial

**DOI:** 10.2196/93352

**Published:** 2026-08-21

**Authors:** Elinny Raissa Moreira dos Reis, Gabriela Martins Alencar, Amanda Maria Schneider, Mari Claussen Tani Fernandes, Juliana de Melo Batista dos Santos, Celso Ricardo Fernandes Carvalho

**Affiliations:** 1 Department of Physical Therapy University of São Paulo School of Medicine São Paulo, SP Brazil; 2 Marketing Department, VitalAire São Paulo, São Paulo Brazil; 3 Air Liquide (France) Paris, Île-de-France France; 4 Department of Health Science Santo Amaro University São Paulo, São Paulo Brazil

**Keywords:** health-related quality of life, HRQoL, quality of life, chronic obstructive pulmonary disease, COPD, interstitial lung disease, ILD, remote monitoring, long-term oxygen therapy, LTOT, randomized controlled trial, RCT

## Abstract

**Background:**

Individuals receiving long-term oxygen therapy (LTOT) are often confined to their homes and have impaired health-related quality of life (HRQoL). Despite technological advances, monitoring these individuals remains a challenge.

**Objective:**

This study aims to evaluate the impact of remote monitoring on HRQoL, dyspnea, health status, symptoms of anxiety and depression, and adherence to data entry via a mobile app in individuals receiving LTOT.

**Methods:**

This randomized controlled trial will recruit 60 participants, including clinically stable adults diagnosed with chronic obstructive pulmonary disease (COPD) or interstitial lung disease (ILD) who use LTOT. Participants will be randomized in a 1:1 ratio to either the control group (CG) or the intervention group (IG), stratified by diagnosis (COPD or ILD), and will be followed for 90 days. All participants will use a mobile app that serves as the central platform for remote monitoring. The app enables daily recording of physiological data, including heart rate (HR), peripheral oxygen saturation (SpO_2_), and oxygen flow rate. In addition, validated questionnaires (EQ-5D-5L, modified Medical Research Council [mMRC] Dyspnea Scale, Hospital Anxiety and Depression Scale [HADS], and COPD Assessment Test [CAT] or the King Brief Interstitial Lung Disease [K-BILD] questionnaire, depending on diagnosis) will be self-administered through the app at predefined time points. The CG will receive monthly telephone follow-up (every 30 days), while the IG will receive biweekly telephone follow-up (every 15 days). During these structured calls, trained health care professionals will collect clinical information and ensure data completeness. In addition, participants in the IG will use a wearable smartwatch that enables continuous physiological monitoring (HR and SpO_2_), with data transmitted to a secure platform and analyzed weekly using an AI-assisted system to support adherence monitoring and identify physiological patterns. Participants in the IG may receive additional contact if clinically relevant abnormalities or reduced adherence are detected. Outcomes will be interpreted according to the minimal clinically important difference (MCID) for dyspnea (mMRC Dyspnea Scale), health status (CAT or K-BILD), quality of life (EQ-5D-5L), and psychological symptoms (HADS).

**Results:**

Funding and ethics approval were obtained in April 2023. Recruitment began in January 2024 and was completed in May 2026. A total of 62 participants were randomized, of whom 60 initiated follow-up and were included in the longitudinal analysis. Overall, 49 participants completed the 90-day follow-up. Data collection has been completed, and data analysis is ongoing. The results are expected to be submitted for publication in the first half of 2027.

**Conclusions:**

This study will provide relevant evidence on the effect of remote monitoring in individuals receiving LTOT on HRQoL, dyspnea, health status, anxiety and depression, and engagement with data entry through a mobile app.

**Trial Registration:**

ClinicalTrials.gov NCT06882265; https://clinicaltrials.gov/study/NCT06882265

## Introduction

Hypoxemia is common in individuals with respiratory diseases and can represent a potentially harmful physiological impairment associated with adverse outcomes [1]. Additionally, individuals with hypoxemia often experience persistent dyspnea, reduced physical activity, and social isolation [2], leading to impaired health-related quality of life (HRQoL) [3]. Long-term oxygen therapy (LTOT) is recommended for chronic hypoxemia and involves administering oxygen for at least 15 hours per day.

According to clinical guidelines, LTOT is indicated for patients with chronic respiratory diseases [4]. LTOT has positive effects on fatigue, sleep quality, and physical activity and increases survival time [5]. Despite these benefits, managing LTOT remains challenging due to the need to monitor correct oxygen use, adjust the oxygen dose based on clinical progression, address device-related limitations, and ensure patient adherence to therapy [5-7]. These challenges highlight opportunities to improve and optimize monitoring strategies and have driven interest in remote monitoring technologies as a promising approach to support patients receiving LTOT.

Remote monitoring can allow continuous or periodic tracking of oxygen saturation, respiratory rate, and patient activity levels. These data may support clinical decision-making and enhance patient management [8]. However, few studies have evaluated the clinical outcomes of remote monitoring specifically in patients receiving LTOT, highlighting the need for further research in this population [9-11]. Furthermore, we hypothesize that remote monitoring would improve the quality of life of patients receiving LTOT.

This study aims to evaluate the impact of remote monitoring with wearable devices on HRQoL in individuals receiving LTOT. The secondary aim is to assess the effects of remote monitoring on psychosocial status, dyspnea severity, and adherence to data entry via a mobile app.

## Methods

### Participants

Individuals of either sex aged 18 to 85 years with chronic hypoxemia due to chronic obstructive pulmonary disease (COPD) or interstitial lung disease (ILD) will be eligible if they have been receiving LTOT for at least 6 months, are receiving optimized pharmacological treatment, and have had no exacerbations during the preceding 2 months. They also need to own a smartphone compatible with the app that monitors quality of life and vital signs. Exclusion criteria will include cognitive or other limitations that impair the understanding of questionnaires, residence outside the metropolitan area where the hospital is located, and inability to use a smartphone or use of a device with an operating system other than iOS or Android. Participants will be screened and recruited at the pulmonology outpatient clinic of a tertiary university hospital. Enrollment in the study will occur after the informed consent form is signed. The schedule of enrollment, allocation, interventions, and assessments is outlined in Table 1.

**Table 1:** Schedule of study assessments and data collection during the 90-day follow-up period.

Assessments	Screening	Baseline (day 0)	Day 15	Day 30	Day 45	Day 60	Day 75	Day 90
Eligibility screening	✓							
Informed consent	✓							
Randomization		✓						
Mobile app installation and training		✓						
Control group telephone follow-up				✓		✓		✓
Intervention group telephone follow-up			✓	✓	✓	✓	✓	✓
Wearable monitoring (intervention group)		✓	✓	✓	✓	✓	✓	✓
Daily physiological data entry via the mobile app^a^		✓	✓	✓	✓	✓	✓	✓
EQ-5D-5L		✓		✓		✓		✓
Hospital Anxiety and Depression Scale		✓		✓		✓		✓
Modified Medical Research Council Dyspnea Scale		✓		✓		✓		✓
COPD Assessment Test or King Brief Interstitial Lung Disease questionnaire		✓		✓		✓		✓

^a^Participants are instructed to enter physiological data into the mobile app daily throughout the follow-up period.

### Study Design

**Figure 1:**
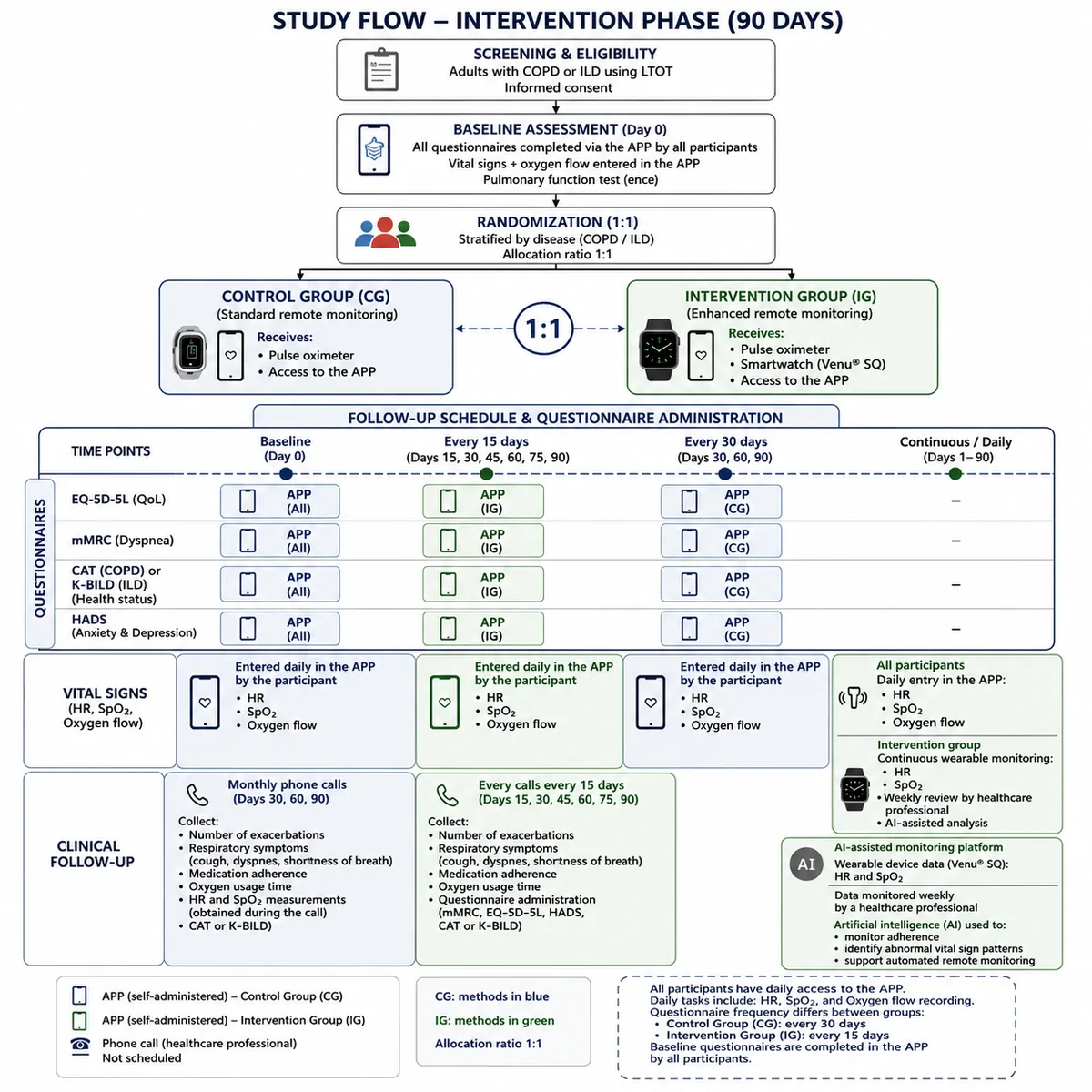
Study design. AI: artificial intelligence; APP: application; CAT: COPD Assessment Test; CG: control group; COPD: chronic obstructive pulmonary disease; HADS: Hospital Anxiety and Depression Scale; HR: heart rate; IG: intervention group; ILD: interstitial lung disease; K-BILD: King Brief Interstitial Lung Disease questionnaire; LTOT: long-term oxygen therapy; mMRC: modified Medical Research Council Dyspnea Scale; QoL: quality of life; SpO₂: peripheral oxygen saturation.

This randomized, controlled, 2-arm, assessor-blinded clinical trial protocol was approved by the Hospital das Clínicas, University of São Paulo Medical School research ethics committee (6.012.012) and registered at ClinicalTrials.gov (NCT06882265). Adults receiving LTOT will be invited to participate in the study after a pulmonology outpatient consultation. Participants will be assessed and followed for 90 days. At the initial visit, all participants will undergo the following assessments: anthropometric measurements (weight and height) and clinical characteristics, including the number of exacerbations during the 6 months prior to randomization, respiratory symptoms, medication use, and daily oxygen therapy use and prescription. Patients were not involved in the design, conduct, reporting, or dissemination plans of this study. The study protocol was developed in accordance with the SPIRIT (Standard Protocol Items: Recommendations for Interventional Trials) checklist guidelines [12], and the study design is presented in Figure 1.

### Mobile App and Baseline Assessments

A study-specific mobile app will be installed on participants’ smartphones and used as the central platform for remote monitoring. The app allows daily recording of physiological data, including heart rate (HR), peripheral oxygen saturation (SpO_2_), and oxygen flow rate. In addition, validated questionnaires (EQ-5D-5L, modified Medical Research Council [mMRC] Dyspnea Scale, Hospital Anxiety and Depression Scale [HADS], and COPD Assessment Test [CAT] or King Brief Interstitial Lung Disease [K-BILD] questionnaire, depending on diagnosis) will be self-administered through the app at predefined time points during follow-up. At baseline, all participants will complete the same standardized questionnaires through the app prior to randomization.

All participants will receive standardized training on the use of the pulse oximeter and the mobile app, as well as a written instruction manual. Participants in the intervention group (IG) will also receive detailed guidance on synchronizing the wearable device with the smartphone app.

### Randomization and Blinding

Participants will be randomized in a 1:1 ratio to either the control group (CG) or the IG. The randomization sequence will be generated using an online randomizer (Sealed Envelope; Sealed Envelope Ltd) by an investigator who is not involved in patient recruitment, assessment, or intervention and who will perform the group allocation. Group allocation will be concealed using consecutively numbered, sealed, opaque envelopes. Randomization will be stratified by diagnosis (ILD or COPD).

### Interventions

#### Control Group

Participants in the CG will receive a pulse oximeter (Morya, model AS304) and will be followed for 90 days. They will receive monthly telephone follow-up every 30 days. During these structured calls, a trained health care professional will collect clinical information, including exacerbations, respiratory symptoms (cough, dyspnea, and shortness of breath), medication adherence, and oxygen use. All study questionnaires (EQ-5D-5L, mMRC Dyspnea Scale, HADS, and CAT or K-BILD, depending on diagnosis) will be self-administered by participants through the mobile app at predefined time points (baseline, day 30, day 60, and day 90). During scheduled telephone follow-up calls, health care professionals will reinforce questionnaire completion, collect clinical information (exacerbations, respiratory symptoms, medication adherence, and oxygen use), and provide support as needed. HR and SpO_2_ will also be recorded using the pulse oximeter on the day of follow-up. Participants will continue using the mobile app for daily recording of physiological data.

#### Intervention Group

Participants in the IG will receive a pulse oximeter and the Garmin Ltd Venu SQ Smartwatch (Garmin International Inc; 2020 wearable monitor). The wearable device will be synchronized with the mobile app, enabling the continuous transmission of HR and SpO_2_ data to a secure cloud-based platform. Oxygen flow rate will be entered by participants through the mobile app.

The IG will receive biweekly telephone follow-up every 15 days. All study questionnaires (EQ-5D-5L, mMRC Dyspnea Scale, HADS, and CAT or K-BILD, depending on diagnosis) will be self-administered by participants through the mobile app at predefined time points (baseline, day 30, day 60, and day 90). During the scheduled telephone follow-up calls, health care professionals will reinforce questionnaire completion, collect clinical information (exacerbations, respiratory symptoms, medication adherence, and oxygen use), and provide support as needed. In addition, wearable-derived data will be monitored weekly by the research team and analyzed using an AI-assisted system designed to identify physiological patterns and support adherence monitoring. The system does not perform autonomous clinical decision-making; all alerts are reviewed by health care professionals prior to any clinical action.

The remote monitoring platform includes an automated data analysis system designed to support adherence monitoring and the identification of abnormal physiological patterns. The system analyzes daily patient-entered data and wearable device data, including HR, SpO_2_, oxygen flow, and the frequency of data entry. The automated analysis system applies predefined rules based on individual baseline values established during the first 7 days of monitoring. Alert criteria include (1) sustained SpO_2_ reduction of ≥4% below the individual baseline; (2) sustained HR variation of 20 beats per minute more or less than the individual baseline; and (3) absence of data entry for 2 consecutive days, defined as low adherence. When any of these criteria are met, the system generates an alert. Although the platform automatically identifies predefined alert patterns, it does not perform autonomous clinical decision-making. All alerts are reviewed and interpreted by a trained health care professional within 24 hours before any participant contact or clinical recommendation. If the alert is confirmed as clinically relevant, the participant is contacted by telephone and, depending on the severity, advised to maintain self-care, contact their usual health care provider, or seek immediate medical evaluation. The remote monitoring system is intended to support patient monitoring and does not replace routine clinical care. Participants are also instructed to contact their usual health care provider whenever necessary.

Both groups receive enhanced standard care. However, the IG will receive continuous wearable-based physiological monitoring and more frequent follow-up, allowing the evaluation of the incremental benefit of remote monitoring beyond standard care. The primary purpose of these telephone contacts is data collection rather than active safety monitoring. However, any clinically relevant concerns identified during the calls will be appropriately addressed and, if necessary, escalated to ensure participant safety.

### Outcomes

#### Primary Outcome: HRQoL

The EuroQol questionnaire (EQ-5D-5L) is a tool used to assess HRQoL, comprising 1 question for each of the 5 dimensions: mobility, self-care, usual activities, pain or discomfort, and anxiety or depression. Responses can be converted into 3125 unique health states or converted into EQ-5D index values using an appropriate value set, typically anchored at 1 for perfect health and 0 for death, with possible negative values for health states considered worse than death. The EQ-5D questionnaire also includes a visual analog scale (VAS), through which respondents can report their perceived health status, with scores ranging from 0 (the worst possible health state) to 100 (the best possible health state). The minimal clinically important difference (MCID) for the EQ-5D-5L is not fixed and may vary according to baseline health status, population characteristics, and the type of intervention. Therefore, the interpretation of clinically meaningful change will consider context-specific estimates reported in the literature [13].

#### Secondary Outcomes

##### Anxiety and Depression Symptoms

The HADS will assess anxiety and depression symptoms. The questionnaire was developed to identify symptoms of anxiety and depressive mood and has been translated, validated, and published in Portuguese [14]. It consists of 14 multiple-choice questions divided into 2 subscales, HADS-anxiety (HADS-A) and HADS-depression (HADS-D), with 7 questions each. The domains are categorized by symptom severity: 0-7=“none,” 8-10=“probable,” and ≥11=“present” [15]. A change of 1.3 points in the anxiety domain and 1.5 points in the depression domain is considered the MCID [16].

##### Health Status

Health status in patients with COPD will be assessed using the CAT, a disease-specific questionnaire that evaluates the impact of symptoms on health status. It consists of 8 items related to health conditions and has been validated in several languages, including Portuguese [17]. A change of 2.0 points is considered the MCID [18].

Health status in patients with ILD will be assessed using the K-BILD questionnaire, a disease-specific instrument developed and validated for patients with ILD. It consists of 15 items that assess health status over the previous 2 weeks across 3 domains: psychological status, breathlessness and activities, and chest symptoms. The questionnaire is easy to administer and understand. Scores range from 0 to 100, with higher scores indicating better health status. A change of 5.4 points in the psychological domain, 4.4 points in the breathlessness domain, 3.9 points in the chest symptoms domain, and 3.9 points in the total score is considered the MCID [19]. The K-BILD has been translated and culturally adapted into Portuguese for use in Brazilian patients [20].

##### Dyspnea Symptoms

Dyspnea will be assessed using the mMRC Dyspnea Scale. It is a unidirectional scale ranging from 0 to 4 points that assesses daily activities that cause dyspnea [21]. A change of 1.0 point is considered the MCID [22].

##### Patient Adherence

Adherence will be assessed based on data entry into the mobile app. A participant will be considered adherent if data (HR, oxygen saturation, and oxygen flow) are entered for at least 30 of the 90 days of follow-up [23,24]. This threshold was defined to ensure a minimum level of engagement with the monitoring system while accounting for occasional missed entries.

### Statistical Analysis

The sample size calculation, based on the primary outcome of the study (EQ-5D-5L), was informed by a previous study reporting an observed difference of 0.04 in the EQ-5D-5L index, which was considered clinically meaningful within that specific study population [13]. This value was used as a pragmatic estimate of the expected effect size for sample size determination. The calculation was performed using G*Power software (*F* tests: ANOVA, repeated measures, within-between interaction). The calculation assumed an effect size of *f*=0.25, a 2-sided α level of .05, and a statistical power of 90% (β=.10). The design included 2 groups and 4 repeated measurements, with a correlation among repeated measures of 0.5 and a nonsphericity correction (ε) of 0.5. On the basis of these assumptions, the required sample size was 50 participants (25 per group), with an achieved power of 0.91. Considering an anticipated dropout rate of 20%, the final target sample size was increased to 30 participants per group to preserve statistical power under an intention-to-treat framework. The significance level will be set at 5% (*P*<.05) for all statistical tests, which will be conducted using GraphPad Prism (version 5.0; GraphPad Software LLC).

Data normality will be assessed using the Kolmogorov-Smirnov test, and homoscedasticity will be evaluated using the Levene test. Parametric data, defined by a normal distribution as demonstrated by histograms, will be presented as means and SDs (or SEs), while nonparametric variables will be presented as medians and IQRs.

Longitudinal outcomes will be analyzed using linear mixed-effects models with fixed effects for group, time, and group-by-time interaction and random intercepts for participants. The results will be interpreted in light of the established MCIDs for each outcome measure. Additionally, as a complementary analysis, the proportion of participants in each group achieving a within-individual change equal to or greater than the MCID will be calculated, providing a more clinically meaningful interpretation of the findings. Sensitivity analyses will be conducted to assess the robustness of the results.

The adherence criterion will be used in exploratory subgroup analyses to compare outcomes between adherent and nonadherent participants [25]. All primary longitudinal analyses will follow a modified intention-to-treat approach, including randomized participants who initiated follow-up and contributed data to the longitudinal analysis, regardless of adherence status. This approach preserves the benefits of randomization and reflects real-world conditions. Sensitivity analyses will be conducted to assess the robustness of the results.

For exploratory purposes, when appropriate, comparisons between groups at specific time points may be performed using parametric or nonparametric tests (eg, the *t* test or Mann-Whitney test), depending on data distribution. Pearson correlation coefficients will be used to assess associations between normally distributed variables, while Spearman correlation coefficients will be used for nonnormally distributed variables.

### Ethical Considerations

The study has already been approved by the Hospital das Clínicas, University of São Paulo Medical School research ethics committee (6.012.012) and registered at ClinicalTrials.gov (NCT06882265). Any important protocol modifications will be submitted to the research ethics committee and updated on ClinicalTrials.gov. All participants will receive verbal and written information about the study and will provide signed informed consent before participating. Information collected from participants is accessible only to the research team and is individually identifiable by a unique numeric ID code. All electronic data are stored using this code, ensuring that no identifiable information is retained until study completion. Confidential information is stored in locked filing cabinets, and only deidentified data will be presented or published. A formal data monitoring committee was not considered necessary due to the low-risk nature of the intervention and the short follow-up period. Trial conduct and data quality will be monitored by the research team throughout the study.

## Results

Funding and ethics approval were obtained in April 2023. Recruitment began in January 2024 and was completed in May 2026. A total of 62 participants were randomized, of whom 60 initiated follow-up and were included in the longitudinal analysis. Overall, 49 participants completed the 90-day follow-up. Data collection has been completed, and data analysis is ongoing. The results are expected to be submitted for publication in the first half of 2027.

## Discussion

Individuals receiving LTOT represent a particularly vulnerable subgroup of patients with chronic respiratory diseases. They often experience severe functional limitations, reduced autonomy, and high rates of health care use, making them an ideal target population for interventions aimed at improving self-management and HRQoL [26]. ILD and COPD account for most LTOT prescriptions and are associated with a high symptom burden and progressive clinical decline [27].

Moreover, among patients with chronic respiratory diseases, especially those with COPD and ILD, heterogeneity in beliefs and perceptions about the disease can significantly influence symptoms, quality of life, and self-management capacity. In addition, frequent remote monitoring may negatively affect emotional well-being in some individuals by increasing health-related anxiety or symptom awareness. Therefore, assessing anxiety and depression symptoms using the HADS may provide important information regarding the psychological impact of remote monitoring in this population. An analysis conducted among patients with COPD identified 2 distinct profiles: a “distressed” group characterized by a higher emotional burden, worse HRQoL, increased dyspnea, and lower self-efficacy; and a “coping” group with more adaptive perceptions. Despite similar levels of lung function and physical activity, these profiles showed relevant clinical and psychosocial differences, highlighting the importance of routinely assessing disease perceptions to personalize interventions and optimize clinical outcomes [28]. In this context, remote monitoring appears to be a suitable method for following patients receiving LTOT, as it enables continuous tracking of parameters such as oxygen saturation, respiratory rate, and physical activity, facilitating the early detection of clinical decompensation. These technologies encourage self-care by increasing patients’ awareness of their condition, which is essential for improving treatment adherence and quality of life [29,30]. However, longer follow-up periods may be necessary to detect more consistent and clinically meaningful changes in HRQoL. Despite these potential benefits, remote monitoring faces significant challenges. The heterogeneity of available devices and the need for adequate technological infrastructure are barriers to the widespread adoption of this technology [10]. Additionally, adherence to LTOT and patients’ perceptions of oxygen therapy may vary and may influence the effectiveness of home-based monitoring [31,32].

If the study findings confirm our hypothesis, they will align with previous research indicating that digital health interventions can enhance patient engagement and emotional well-being by providing a sense of connection and safety within the home environment. Few studies have evaluated the clinical outcomes of remote monitoring specifically in patients receiving LTOT, highlighting the need for further research in this population. Therefore, this study seeks to provide evidence to support the safe implementation of home care services, and the results are expected to guide clinical practice and future home care strategies.

This study has some anticipated limitations. The sample size may not have adequate statistical power for the secondary outcomes. Although the follow-up period may be considered short, it should be sufficient to detect changes in quality of life and assess participants’ adherence. The frequency of data collection differed between groups, reflecting the distinct nature of the approaches: the CG performed self-monitoring, whereas the IG received more intensive remote monitoring. However, this difference may influence outcomes such as adherence and health perception. On the other hand, this design makes the comparison more conservative and closer to real-world clinical practice, allowing an evaluation of the incremental value of remote monitoring over standard care.

The potential clinical implications of this research include validating remote monitoring as an effective tool for optimizing the management of home oxygen therapy. This approach may enable faster and more personalized interventions, promote greater patient autonomy, and improve quality of life, thereby contributing to better health outcomes.

## Data Availability

The datasets used and/or analyzed during the study are available from the corresponding author upon reasonable request.

## Appendix

Multimedia Appendix 1 SPIRIT checklist.

## Abbreviations

CAT: COPD Assessment Test
CG: control group
COPD: chronic obstructive pulmonary disease
HADS: Hospital Anxiety and Depression Scale
HADS-A: Hospital Anxiety and Depression Scale–anxiety
HADS-D: Hospital Anxiety and Depression Scale–depression
HR: heart rate
HRQoL: health-related quality of life
IG: intervention group
ILD: interstitial lung disease
K-BILD: King Brief Interstitial Lung Disease
LTOT: long-term oxygen therapy
MCID: minimal clinically important difference
mMRC: modified Medical Research Council
SPIRIT: Standard Protocol Items: Recommendations for Interventional Trials
SpO2: peripheral oxygen saturation
VAS: visual analog scale
